# Diabetes complications in people with alcohol use disorder and type 2 diabetes

**DOI:** 10.3399/BJGPO.2024.0133

**Published:** 2025-06-04

**Authors:** Sarah Cook, Sonia Saxena, Rohini Mathur, Thomas Beaney, Shamini Gnani, Ana Luisa Neves, Arti Maini, Ravi Parekh, Kate Walters, David Osborn, Jennifer K Quint

**Affiliations:** 1 School of Public Health, Imperial College London, London, UK; 2 Wolfson Institute of Population Health, Queen Mary University of London, London, UK; 3 Primary Care and Population Health, University College London, London, UK; 4 Division of Psychiatry, University College London, London, UK; 5 Camden and Islington NHS Foundation Trust, London, UK

**Keywords:** Comorbidity, Diabetes, Addictions

## Abstract

**Background:**

People living with alcohol use disorder (AUD) who develop type 2 diabetes (T2DM) may be at higher risk of diabetes complications.

**Aim:**

Our aim was to compare diabetes monitoring and incidence of diabetes complications between people with and without AUD prior to T2DM diagnosis attending primary care in England.

**Design & setting:**

We used the Clinical Practice Research Datalink Aurum linked with Hospital Episode Statistics and Office for National Statistics mortality data. The target population was people with incident T2DM diagnosed between 2004 and 2019.

**Method:**

We defined AUD from codes indicating i) clinical diagnosis; ii) alcohol withdrawal; or iii) chronic alcohol-related harm. Outcomes were end-stage renal disease (ESRD), lower limb amputation, myocardial infarction (MI), stroke, cardiovascular disease (CVD) mortality, and all-cause mortality. We compared yearly HbA1c, creatinine, and cholesterol monitoring activities for the first 5 years post T2DM diagnosis.

**Results:**

The study population was 543 509 people, of whom 15 237 (2.8%) had a code for AUD. Adjusting for measured confounders, people with AUD had higher rates of ESRD ( incidence rate ratio [IRR] 1.95, 95% confidence intervals [CI] = 1.71 to 2.23), lower limb amputation (IRR 1.78, 95% CI = 1.50 to 2.21), stroke (IRR 1.36, 95% CI = 1.25 to 1.47), CVD mortality (IRR 1.74, 95% CI = 1.63 to 1.86), and all-cause mortality (IRR 2.10, 95% CI = 2.04 to 2.17) but not MI (IRR 0.91, 95% CI = 0.82 to 1.00) compared with people without AUD. Laboratory diabetes monitoring was high in people with (83.5–91.1%) and without (83.7–92.4%) AUD.

**Conclusion:**

People with AUD had nearly double the rates of most of the diabetes complications investigated compared with people without AUD.

## How this fits in

People living with alcohol use disorder (AUD) who develop type 2 diabetes (T2DM) may experience more challenges with disease management than people without AUD and be at higher risk of diabetes complications. Two previous studies, both using electronic health record data from the US, have investigated the relationship between AUD and diabetes complications with contrasting results. To our knowledge, this relationship has not been investigated in other countries with different health services to the US. In a primary care population in England we found that people with AUD at the time of T2DM diagnosis were more likely to experience diabetes complications, with 40% higher rates of stroke and nearly double the rates of end-stage renal disease, lower limb amputation, cardiovascular disease mortality, and all-cause mortality. T2DM laboratory blood test monitoring in primary care did not explain this.

## Introduction

AUD is a serious public health concern affecting approximately 8.6% of adult men and 1.7% of adult women worldwide.^1^ People with AUD have more than two times higher all-cause mortality compared with the general population,^2^ and are five times more likely to die from diabetes.^3^

People living with AUD who develop a chronic disease may experience more challenges with disease management than people without AUD. For T2DM, where management is complex and requires a high level of interaction with health services, this may translate to worse health outcomes. If not well managed, T2DM can lead to a range of complications, the consequences of which can be very severe, including amputation, end-stage renal disease (ESRD), cardiovascular disease (CVD), and mortality.^4,5^

Two previous studies using electronic health record (EHR) data from the US have investigated the relationship between AUD and diabetes complications with contrasting results. A matched cohort study including 8120 people with T2DM and hypertension from Ohio found that people who also had AUD had 27% higher odds of diabetic neuropathy, 62% higher odds of myocardial infarction (MI), and 54% higher odds of all-cause mortality, but no higher odds of stroke or diabetic renal disease.^6^ In contrast, a cohort study of 106 174 people in Massachusetts, who were beneficiaries of Medicare or Medicaid, found that people with AUD had 53% higher odds of lower limb amputation and 26% higher odds of cerebrovascular disease, but lower odds of eye complications and diabetic neuropathy, and no difference in odds of nephropathy, diabetes-related hospitalisation, or ischaemic heart disease.^7^ To our knowledge, the relationship between AUD and diabetes complications has not been investigated in countries with different health services to the US. If differential access to health services increases the risk of some complications, these differences could be expected to be smaller in countries that have a national health service, such as in the UK, where health care is free at the point of delivery.

There is some evidence that AUD may impact levels of diabetes monitoring. A US EHR study found lower levels of some diabetes monitoring activities (low-density lipoprotein cholesterol and eye screening) among people with diabetes and AUD, although HbA1c monitoring levels in this study were the same.^8^ We hypothesised that differential uptake of diabetes monitoring among people with AUD may contribute to differences in the risk of developing diabetes complications.

The aims of this study were to compare the incidence of T2DM complications between people with and without prior AUD in primary care in England and to investigate whether diabetes monitoring mitigated any differences in complication rates between those with and without AUD.

## Method

### Setting and data sources

We used data from the Clinical Practice Research Datalink (CPRD) Aurum.^9,10^ The CPRD Aurum contains EHR data collected during routine health care from a sample of primary care practices in the UK which use EMIS Web general practice patient management software. This includes information about demographic characteristics, diagnoses and symptoms, medication prescriptions, and laboratory tests. The CPRD Aurum population is representative of the UK population in terms of age, gender, geographic location, and deprivation.^9^ The May 2022 version of the database used for this study covered 19.83% of the UK population.^10^

CPRD Aurum data were linked with Hospital Episode Statistics Admitted Patient Care (HES APC),^11^ Index of Multiple Deprivation (IMD),^12^ and Office for National Statistics (ONS) mortality data.^13^

HES contains data on patients admitted to NHS hospitals in England. As eligibility for linkage with HES data was a requirement for this study, we restricted our study population to people resident in England rather than the whole of the UK.

### Participants

**Figure 1.**
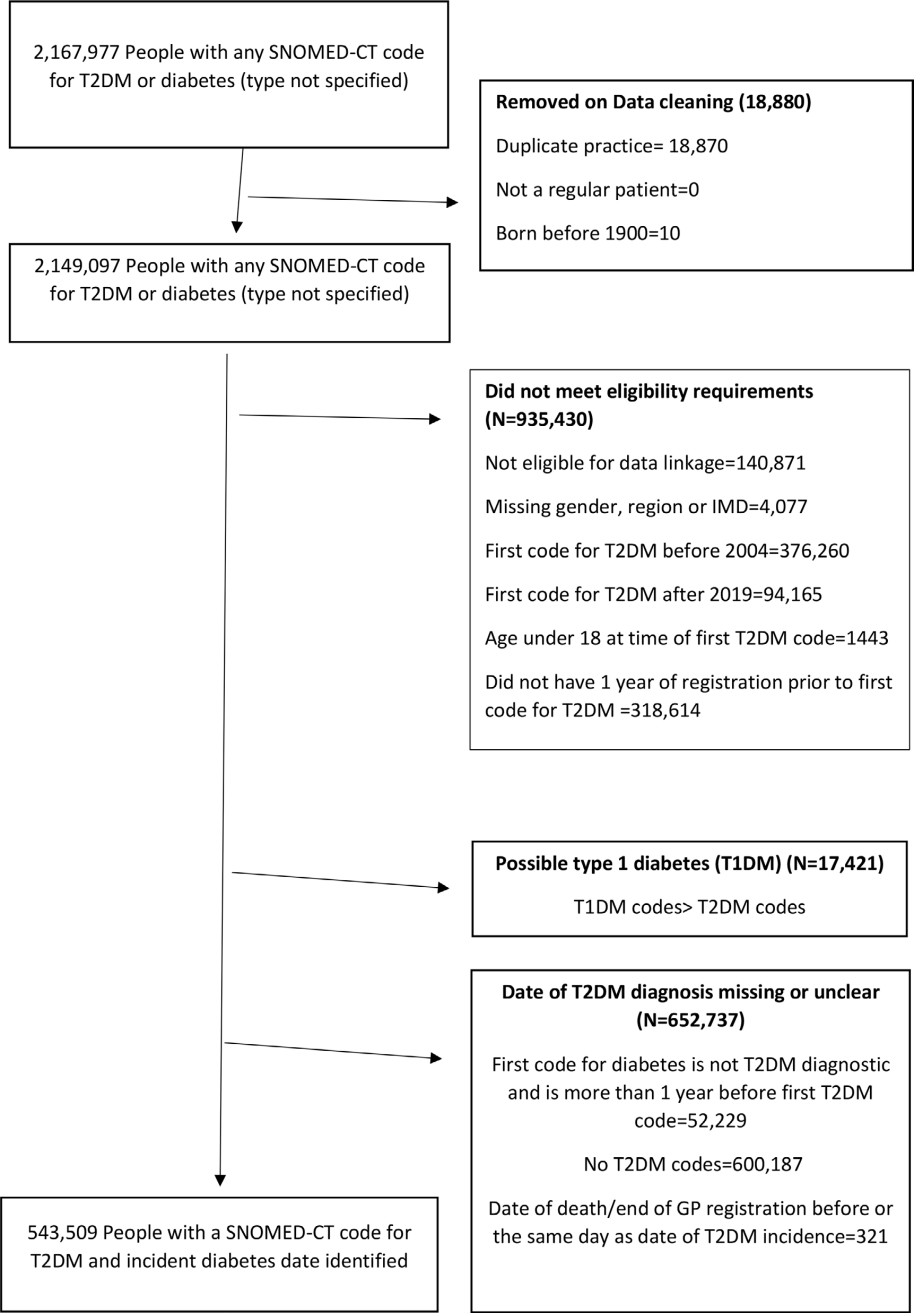
Selection of people with incident type 2 diabetes (T2DM) between 2004 and 2019 IMD = Index of Multiple Deprivation.

The target population was people with incident T2DM between 1 January 2004 and 1 January 2020. We defined date of T2DM incidence as the first date for a T2DM diagnosis or a T2DM-related code from a broader code list of diabetes SNOMED CT codes (https://github.com/NHLI-Respiratory-Epi/diabetes_alcohol_use_disorder/blob/main/diabetes_broad_list_vs3.xls). Exclusion criteria are shown in Figure 1.

Codelists used for deriving the T2DM cohort and all other variables used for these analyses are available on Github (https://github.com/NHLI-Respiratory-Epi/diabetes_alcohol_use_disorder).

### Study variables

We adapted the International Classification of Diseases 10 (ICD-10) definition of AUD to capture diagnoses of alcohol dependence syndrome and harmful alcohol use as defined in Chapter F10.1–F10.9 Mental and Behavioural disorders due to Alcohol.^14,15^ In line with this definition, AUD was defined as presence of a SNOMED-CT code in the primary care record which indicated i) clinical diagnosis; or ii) alcohol withdrawal; or iii) chronic alcohol-related harm to health (physical or mental); or any ICD-10 code from F10.1–F10.9 from the HES APC records prior to T2DM diagnosis.

High levels of blood glucose over time can lead to damage to the small blood vessels (microvascular complications) and to the larger blood vessels (macrovascular complications). We included a range of outcomes including both microvascular and macrovascular complications which were likely to be well recorded in EHR data.

Diabetes complications included as outcomes were:

1. ESRD as indicated by initiation of chronic dialysis or renal transplantation defined either in primary or secondary care.
2. Amputations of the lower limb (leg, foot, or toe). Amputation was defined from HES APC data using the Office of Population Censuses and Surveys’ Classification of Surgical Operations and Procedures, 4th revision (OPCS-4) codes (X9, X10, and X11)^16^
3. First episode MI defined as the first SNOMED-CT code in primary care or first ICD-10 code as the primary diagnosis for hospital admission post T2DM diagnosis
4. First episode stroke defined as the first SNOMED-CT code in primary care or first ICD-10 code as the primary diagnosis for hospital admission post T2DM diagnosis
5. CVD mortality defined using death date and underlying cause of death recorded by ONS
6. All-cause mortality defined using death date from ONS death records.

Confounding factors included in the model were:

- Socio-demographic factors (age at T2DM diagnosis, current age, gender, region, ethnicity, and quintiles of area-level socioeconomic status measured by IMD)
- Calendar time (year of T2DM diagnosis and calendar period)
- Smoking status
- Body mass index
- Physical health conditions defined as number of comorbidities using the Charlson Comorbidity Index,^17^ excluding diabetes
- Mental health condition (depression, anxiety, and severe mental illness).

We investigated three indicators of yearly diabetes monitoring in the first 5 years post T2DM diagnosis: HbA1c, serum creatinine, and total cholesterol. These were considered present if there was a code recorded, regardless of whether measurement values were recorded or valid.

### Statistical analysis

Separate Poisson regression models were fitted for each outcome, adjusting for a) age, gender, calendar time, age and year of T2DM diagnosis; and b) all measured confounders. People with the outcome prior to T2DM diagnosis were excluded for that specific analysis. In sensitivity analysis, models were fitted using the AUD variable split by whether coding suggested people were currently drinking or not at the time of T2DM diagnosis. The ‘not currently drinking‘ group was defined from 1) a SNOMED-CT code in primary care for non-drinking more recent than their most recent code for AUD; or 2) most recent AUD SNOMED-CT code in primary care indicated remission; or 3) no AUD codes in primary or secondary care in the past 5 years.

Descriptive analysis was conducted comparing the proportion of people each year with codes for i) HbA1c, ii) serum creatinine, and iii) total cholesterol by whether people had a code for AUD prior to T2DM diabetes diagnosis for the first 5 years post T2DM diagnosis using χ^2^ tests. The denominator for each year was restricted to people in follow-up at that time.

Variables indicating whether each laboratory test was measured in the first year post T2DM diagnosis were added to the fully adjusted Poisson regression models to see if this explained any of the relationship between AUD and rate of diabetes complications (were mediators of the relationship).

Complete case analysis was used for regression modelling. Analyses were conducted using Stata (version 17).

Analyses were conducted using Stata (version 17).

## Results

There were 543 509 people with incident T2DM identified between 2004 and 2019. Of these, 15 237 (2.8%) had a previous code for AUD. Follow-up time was 3 145 295 person years for people without codes for AUD and 71 466 person years for people with codes for AUD. Median follow-up time was 5.1 years (interquartile range [IQR] 2.2–9.1 years).

The characteristics of the study population at time of T2DM diagnosis are shown in Table 1. People with a code for AUD prior to T2DM diagnosis were younger at T2DM diagnosis, more likely to be male, to currently smoke, and to have a higher number of co-existing physical and mental health conditions (Table 1).

**Table 1.** Characteristics of people with and without a code for alcohol use disorder (AUD) at time of type 2 diabetes (T2DM) diagnosis

		AUD code recorded prior to T2DM diagnosis		No AUD code prior to T2DM diagnosis	
		*n*	(%)	*n*	(%)
Total		15 237	(100)	528 272	(100)
Age at time of T2DM diagnosis, years	Mean (SD)	57.6	(11.5)	61.4	(14.0)
Gender	Male	11 404	(74.8)	291 730	(55.2)
	Female	3833	(25.2)	236 542	(44.8)
Ethnicity	White	13 892	(91.8)	424 960	(81.9)
	Asian	637	(4.2)	57 770	(11.1)
	Black	385	(2.5)	25 853	(5.0)
	Mixed	106	(0.7)	5124	(1.0)
	Other	106	(0.7)	5426	(1.0)
	Not stated/missing (*% of cohort)	111	(0.7)	9139	(1.7)
Region of England	North East England	741	(4.9)	19 527	(3.7)
	North West England	3932	(25.8)	100 511	(19.0)
	Yorkshire and the Humber	527	(3.5)	19 308	(3.7)
	East Midlands	316	(2.1)	12 573	(2.4)
	West Midlands	2332	(15.3)	97 060	(18.4)
	East of England	494	(3.2)	22 024	(4.2)
	London	2560	(16.8)	98 059	(18.6)
	South East England	2408	(15.8)	96 434	(18.3)
	South West England	1927	(12.6)	62 776	(11.9)
Index of Multiple Deprivation	1 – Least deprived	1741	(11.4)	90 864	(17.2)
	2	2257	(14.8)	99 660	(18.9)
	3	2597	(17.0)	102 455	(19.4)
	4	3540	(23.2)	114 786	(21.7)
	5 – Most deprived	5102	(33.5)	120 507	(22.8)
Year of diagnosis of T2DM	Median (IQR)	2013	(2009, 2017)	2012	(2008, 2016)
Most recent recorded smoking status	Never smoked	1299	(8.6)	113 547	(21.8)
	Used to smoke	7058	(46.9)	318 206	(61.1)
	Currently smokes	6708	(44.5)	89 045	(17.1)
	Missing (*% of cohort)	172	(1.1)	7474	(1.4)
Most recent Body Mass Index	Underweight (<18.5)	238	(1.7)	1905	(0.4)
	Normal (18.5–24.9)	2219	(15.5)	53 614	(10.8)
	Overweight (25–29.9)	4177	(29.1)	154 053	(31.1)
	Obese (≥30)	7704	(53.7)	286 329	(57.7)
	Missing* (% of cohort)	899	(5.9)	32 371	(6.1)
Past depression	Yes	8912	(58.5)	149 602	(28.3)
Past anxiety	Yes	6028	(39.6)	94 219	(17.8)
Severe mental illness	Yes	1639	(10.8)	13 129	(2.5)
Number of comorbidities according to Charlson Comorbidity Index (excluding diabetes)	0	5683	(37.3)	273 737	(51.8)
	1	5310	(34.8)	161 404	(30.6)
	2	2678	(17.6)	60 677	(11.5)
	≥3	1566	(10.3)	32 454	(6.1)
Coding suggests current drinking at the time of diagnosis in people with AUD	Not currently drinking	5225	(34.3)	-	
	Currently drinking	10 012	(65.7)	-	
Source of diagnosis for AUD	Primary care only	6261	(41.1)	-	
	Hospital inpatient record only	4738	(31.1)	-	
	Both hospital and primary care record	4238	(27.8)	-	
Prior MI	Yes	1167	(7.7)	33 608	(6.4)
Prior stroke	Yes	1119	(7.3)	23 670	(4.5)
Prior lower limb amputation	Yes	98	(0.6)	1227	(0.2)
Prior end-stage renal disease	Yes	34	(0.2)	1174	(0.2)

IQR = interquartile range. MI = myocardial infarction.

*People with missing data are not included within the denominator for column percentages

The rates of diabetes complications in people with and without AUD are shown in Table 2.

**Table 2.** Rates of diabetes complications following type 2 diabetes diagnosis in people with and without codes for alcohol use disorder

	Total, *N*	No AUD code			AUD code			Rate difference
		Events	Rate per 1000 person years	95% CI	Events	Rate per 1000 person years	95% CI	
ESRD	541 489	4823	1.54	1.50 to 1.59	266	3.76	3.34 to 4.24	2.22
Lower limb amputation	542 184	3065	0.98	0.94 to 1.01	166	2.34	2.01 to 2.73	1.36
MI	508 734	18 790	6.44	6.35 to 6.53	453	6.91	6.31 to 7.58	0.47
Stroke	518 720	26 308	8.85	8.74 to 8.96	725	11.08	10.30 to 11.92	2.23
CVD mortality	543 509	33 367	10.61	10.50 to 10.72	1089	15.24	14.36 to 16.17	4.63
All-cause mortality		116 861	37.14	36.93 to 37.35	4867	68.10	66.22 to 70.04	30.96

AUD = alcohol use disorder. CVD = cardiovascular disease. ESRD = end-stage renal disease. MI = myocardial infarction.

Adjusting for measured confounders (model 2), people with AUD had higher rates of all outcomes except MI (IRR 0.91, 95% CI = 0.82 to 1.00): ESRD (IRR 1.95, 95% CI = 1.71 to 2.23), lower limb amputation (IRR 1.78, 95% CI = 1.50 to 2.21), stroke (IRR 1.36, 95% CI = 1.25 to 1.47), CVD mortality (IRR 1.74, 95% CI = 1.63 to 1.86), and all-cause mortality (IRR 2.10, 95% CI = 2.04 to 2.17) compared with people without AUD (Table 3).

**Table 3.** Incidence rate ratio for diabetes complications in people with alcohol use disorder (AUD) codes compared with people without AUD codes

	Model 1 (minimal adjustment)^a^		Model 2 (full adjustment)^b^		Model 3 (+indicators of diabetes monitoring)^c^	
	IRR	95% CI	IRR	95% CI	IRR	95% CI
ESRD	2.50	2.19 to 2.84	1.95	1.71 to 2.23	1.94	1.70 to 2.22
Lower limb amputation	2.56	2.16 to 3.03	1.78	1.50 to 2.211	1.77	1.49 to 2.11
MI	1.18	1.07 to 1.30	0.91	0.82 to 1.00	0.90	0.82 to 1.00
Stroke	1.71	1.58 to 1.85	1.36	1.25 to 1.47	1.35	1.25 to 1.46
CVD mortality	2.44	2.29 to 2.61	1.74	1.63 to 1.86	1.73	1.62 to 1.84
All-cause mortality	2.96	2.88 to 3.05	2.10	2.04 to 2.17	2.09	2.02 to 2.15

CVD = cardiovascular disease. ESRD = end-stage renal disease. MI = IRR = incidence rate ratio. MI = myocardial infarction.

abc Age and year of T2DM diagnosis, sex, current age, and calendar time. Age and year of T2DM diagnosis, sex, current age and calendar time, region, IMD quintile, ethnicity, BMI, smoking status, past history of depression, past history of anxiety, past history of severe mental illness, number of comorbidities from Charlson Comorbidity Index. Model 2+HbA1c code in 1^st^ year post T2DM diagnosis+serum creatinine code in 1^st^ year post T2DM diagnosis+total cholesterol code in 1^st^ year post T2DM diagnosis.

After adjusting for all measured confounders (model 2), incidence rate ratios for ESRD were higher in the AUD group likely to be currently drinking (IRR 2.23, 95% CI = 1.92 to 2.60) than in the AUD group who were likely to be not currently drinking (IRR 1.37, 95% CI = 1.06 to 1.79) (*P* = 0.001) (Table 4). The same was observed for all-cause mortality (IRR currently drinking compared with people without AUD 2.33, 95% CI = 2.25 to 2.42; IRR not currently drinking 1.62, 95% CI = 1.45 to 1.81). The association with MI was different in the two AUD groups, with no association with MI in people with AUD with codes suggesting they were not currently drinking compared with no AUD (IRR 1.05, 95% CI = 0.90 to 1.22), while rates of MI were lower in the AUD group who were currently drinking compared with no AUD (IRR 0.83, 95% CI = 0.74 to 0.94).There was no statistical evidence for differences between the AUD groups for lower limb amputation, stroke, and CVD mortality (Table 4).

**Table 4.** Rates and incidence rate ratios (95% CI) for diabetes complications by whether people with alcohol use disorder (AUD) had codes indicating current drinking at time of type 2 diabetes (T2DM) diagnosis

	AUD (Currently drinking)					AUD (Not currently drinking)					*P* value (difference AUD currently vs not currently drinking in fully adjusted model)
	Events	Rate per 1000 person years	95% CI	Model 1 (Minimal adjustment)^a^	Model 2 (Full adjustment)^b^	Events	Rate per 1000 person years	95% CI	Model 1 (Minimal adjustment)^a^	Model 2 (Full adjustment)^b^	
ESRD	204	4.27	3.72 to 4.90	2.89 (2.50 to 3.35)	2.23 (1.92 to 2.60)	62	2.70	2.10 to 3.46	1.73 (1.33 to 2.24)	1.37 (1.06 to 1.79)	*P* = 0.001
Lower limb amputation	112	2.34	1.94 to 2.81	2.52 (2.05 to 3.09)	1.74 (1.41 to 2.14)	54	2.36	1.80 to 3.08	2.62 (1.98 to 3.49)	1.86 (1.40 to 2.48)	*P* = 0.69
MI	282	6.35	5.65 to 7.14	1.09 (0.97 to 1.24)	0.83 (0.74 to 0.94)	171	8.11	6.98 to 9.43	1.34 (1.15 to 1.56)	1.05 (0.90 to 1.22)	*P* = 0.02
Stroke	488	11.02	10.08 to 12.05	1.77 (1.61 to 1.94)	1.40 (1.27 to 1.54)	237	11.21	9.87 to 12.73	1.60 (1.40 to 1.82)	1.28 (1.12 to 1.46)	*P* = 0.26
CVD mortality	734	15.20	14.14 to 16.34	2.54 (2.35 to 2.75)	1.82 (1.67 to 1.95)	355	15.32	13.81 to 17.00	2.27 (2.03 to 2.53)	1.62 (1.45 to 1.81)	*P* = 0.11
All-cause mortality	3499	72.44	70.08 to 74.88	3.31 (3.19 to 3.43)	2.33 (2.25 to 2.42)	1368	59.05	56.00 to 62.27	2.35 (2.23 to 2.49)	1.69 (1.59 to 1.78)	*P*<0.001

CVD = cardiovascular disease. ESRD = end-stage renal disease. MI = myocardial infarction.

ab Age and year of T2DM diagnosis, sex, current age, and calendar time (reference group no codes for AUD). Age and year of T2DM diagnosis, sex, current age and calendar time, region, IMD quintile, ethnicity, BMI, smoking status, past history of depression, past history of anxiety, past history of severe mental illness, number of comorbidities from Charlson co-morbidity index (reference group no codes for AUD).

There were very small differences between people with codes for AUD and people without codes for AUD for yearly diabetes monitoring. These differences increased slightly over the first 5 years following T2DM diagnosis, however, recording of all three laboratory tests was consistently high in everyone (Table 5). Among people without codes for AUD, approximately 90% had at least one HbA1c code each year (approximately 86–87% in people with codes for AUD), approximately 87% had serum creatinine (approximately 85% with AUD codes), and approximately 88% (85% with AUD codes) had total cholesterol codes (Table 5).

**Table 5.** Diabetes laboratory monitoring in the first 5 years following type 2 diabetes diagnosis by alcohol use disorder status

Year since diabetes diagnosis	*N* ^a^	HbA1c code		Serum creatinine code		Cholesterol code		No codes for any laboratory tests	
AUD		*n*	(%)	*n*	(%)	*n*	(%)	*n*	(%)
Year 1	12 648	11 519	(91.1)	10 587	(83.7)	10 566	(83.5)	759	(6.0)
Year 2	10 477	9187	(87.7)	9016	(86.1)	9040	(86.3)	803	(7.7)
Year 3	8762	7575	(86.5)	7439	(84.9)	7430	(84.8)	749	(8.5)
Year 4	7162	6235	(87.1)	6142	(85.8)	6187	(86.4)	538	(7.5)
Year 5	5826	5013	(86.0)	4957	(85.1)	4928	(84.6)	514	(8.8)
No AUD									
Year 1	465 458	430 210	(92.4)	389 434	(83.7)	396 684	(85.2)	23 392	(5.0)
Year 2	408 767	370 095	(90.5)	356 230	(87.1)	362 283	(88.6)	25 943	(6.3)
Year 3	359 494	324 553	(90.3)	313 876	(87.3)	317 757	(88.4)	23 731	(6.6)
Year 4	312 101	281 957	(90.3)	272 932	(87.4)	278 688	(89.3)	19 299	(6.2)
Year 5	269 159	242 966	(90.3)	235 841	(87.6)	237 401	(88.2)	17 638	(6.6)
Difference No AUD - AUD (%)									
Year 1		1.36% (*P*<0.001)		-0.03% (*P* = 0.91)		1.68% (*P*<0.001)		0.97%	
Year 2		2.85% (*P*<0.001)		1.09% (*P* = 0.001)		2.35% (*P*<0.001)		1.31%	
Year 3		3.83% (*P*<0.001)		2.41% (*P*<0.001)		3.59% (*P*<0.001)		1.95%	
Year 4		3.28% (*P*<0.001)		1.69% (*P*<0.001)		2.90% (*P*<0.001)		1.33%	
Year 5		4.22% (*P*<0.001)		2.54% (*P*<0.001)		3.61% (*P*<0.001)		2.27%	

AUD = alcohol use disorder.

a Excluding people after date of death, end of GP registration, last collection date, or end of study (1 Jan 2020).

Further adjustment for laboratory tests (Model 3) in the first year following T2DM diagnosis did not explain the association between AUD and any of the diabetes complications (Table 3).

## Discussion

### Summary

In a primary care population in England, people with AUD at the time of T2DM diagnosis were more likely to experience diabetes complications, with 40% higher rates of stroke and nearly double the rates of ESRD, lower limb amputation, CVD mortality, and all-cause mortality. Diabetes monitoring in primary care was consistently high in people with and without codes for AUD (>83%), and diabetes monitoring in the year post T2DM did not contribute to explaining differences in higher rates of diabetes complications observed. People with AUD with codes suggesting they were not drinking at the time of their T2DM diagnosis had lower rates of ESRD and all-cause mortality.

### Strengths and limitations

CPRD Aurum is a large nationally representative dataset, and we were able to include over 500 000 people with incident T2DM and 15 237 people with AUD, ensuring the study was well powered. However, it is worth also considering that with a very large sample size, small differences between groups may be statistically significant but not clinically relevant in practice. While we found statistical evidence for a difference in diabetes blood monitoring between those with and without AUD, these differences were very small in practice. EHR data are not collected for research purposes and are reliant on what is coded by healthcare practitioners. This is influenced both by coding practices and health service use. Here, we focused on harder end points in order to minimise measurement error. However, that meant we did not consider the full range of diabetes complications, for example diabetic retinopathy, where coding may be related to eye screening practices, or other important indicators of diabetes monitoring such as foot screening. We also did not distinguish between codes for laboratory measurements and whether valid test results were present, therefore there may be some cases where a blood test was requested but not conducted. Our definition of AUD was specific and we have not included people who may be at high risk but did not meet diagnostic criteria for AUD. Conversely, we only included people who were registered with a primary care practice (therefore people experiencing homelessness are much less likely to be included) who had their AUD detected and coded in their healthcare records. These people may be more engaged with health services generally so their levels of diabetes monitoring may be higher than in the whole target population. The categorisation of currently and not currently drinking was pragmatic based on the available data and we cannot say we definitely captured people’s drinking status at T2DM diagnosis, nor have we been able to capture drinking trajectories which change over time.

It was a strength that we were able to adjust for a wide range of confounders, however, residual confounding due to inaccurate or missing data on confounders is still possible. For example, only area-level and not individual-level data on socioeconomic status were available.

### Comparison with existing literature

Our study findings are similar to Leung *et al*.^7^ who found among a cohort study of 106 175 people with T2DM registered with Medicare or Medicaid in Massachusetts in 2004 that people with AUD had 53% higher odds of lower limb amputation and 26% higher odds of cerebrovascular disease in the following year, but no difference in odds of developing ischaemic heart disease. While the effect sizes in our study were higher, these are not directly comparable as Leung *et al*. reported odds ratios rather than rate ratios and a 1 year follow-up (between 2004 and 2005).

Findings from our study and Leung *et al*.^7^ contrast with Winhusen *et al*.^6^ who found in a matched cohort study of 8120 people with T2DM and hypertension that people with AUD had 62% higher odds of MI and 54% higher odds of all-cause mortality but no association with stroke. The reasons for the differences between these studies are not clear but could be due to the difference in study design (matched versus unmatched), follow-up time, and underlying risk in the study populations. Of note, Winhusen *et al*.^6^ included as their target population people with hypertension and T2DM rather than all people with T2DM.

### Implications for research and practice

Our findings have highlighted a substantially increased rate of diabetes complications in people with AUD who develop T2DM. While we hypothesised that lower levels of diabetes monitoring in people with AUD may contribute to a higher risk of diabetes complications, our findings did not support this, at least in terms of blood tests. However, we did not assess quality of interactions between patients and healthcare practitioners, or outcomes in terms of communicating findings to patients and actions taken to improve management if needed. This needs further investigation, especially as our findings suggest that most people with AUD and T2DM attend routine blood tests, representing a potential missed opportunity for intervention.

We found incidence rate ratios for ESRD and all-cause mortality were lower in people with AUD with codes suggesting they were not currently drinking, compared with those with codes suggesting current drinking. Interventions to help people reduce their drinking have been shown to decrease all-cause mortality.^18^ Diagnosis of diabetes can be a strong motivator for behaviour change and offers an opportunity for timely intervention.^19^ Our findings underline the importance of a holistic integrated care approach which includes consideration of the wider context, including alcohol use, for people following diagnosis of T2DM. Here we have identified a substantially higher risk of diabetes complications in people with AUD following T2DM diagnosis, but further work investigating mediators and effect modifiers is needed to better understand mechanisms by which AUD influences outcomes in T2DM. Complementary qualitative research would be valuable to understand how diabetes is managed in people with AUD and what the barriers and supporting factors are in helping people manage both conditions simultaneously. This would help in designing effective targeted interventions and developing resources to improve outcomes for people with AUD and T2DM.

## Data

Data are available on request from the CPRD. Their provision requires the purchase of a licence, and this licence does not permit the authors to make them publicly available to all. This work used data from the version collected in May 2022 and have clearly specified the data selected within each Method section. To allow identical data to be obtained by others, via the purchase of a licence, the code lists will be provided on request. Licences are available from the CPRD (http://www.cprd.com): The Clinical Practice Research Datalink Group, The Medicines and Healthcare products Regulatory Agency, 10 South Colonnade, Canary Wharf, London E14 4PU.

## Public and patient involvement

The study research question and outcomes were discussed with five people with lived experience of alcohol use disorder and diabetes prior to the analysis stage. The findings from the study were shared with and discussed with 23 people with lived experience of alcohol use disorder and diabetes (personal experience or carers/relatives) prior to submitting this manuscript for publication.
